# Discriminating cancer-related and cancer-unrelated chemoradiation-response genes for locally advanced rectal cancers

**DOI:** 10.1038/srep36935

**Published:** 2016-11-15

**Authors:** You Guo, Jun Cheng, Lu Ao, Xiangyu Li, Qingzhou Guan, Juan Zhang, Haidan Yan, Hao Cai, Qiao Gao, Weizhong Jiang, Zheng Guo

**Affiliations:** 1 Department of Bioinformatics, Key Laboratory of Ministry of Education for Gastrointestinal Cancer, Fujian Medical University Fuzhou, China, 350001; 2 Department of Preventive Medicine, School of Basic Medicine Sciences, Gannan Medical University, Ganzhou, China, 341000; 3 Department of Colorectal Surgery, Fujian Medical University Union Hospital, Fuzhou, China, 350001.

## Abstract

For patients with locally advanced rectal cancer (LARC) treated with preoperation chemoradiation (pCRT), identifying differentially expressed (DE) genes between non-responders and responders is a common approach for investigating mechanisms of chemoradiation resistance. However, some of such DE genes might be irrelevant to cancer itself but simply reflect the pharmacokinetic differences of the normal tissues. In this study, we adopted the *RankComp* algorithm to identify DE genes for each of LARC sample compared with its own normal state. Then, we identified genes with significantly different deregulation frequencies between the non-responders and responders, defined as cancer-related pCRT-response genes. Pathway enrichment and protein-protein interaction analyses showed that these genes specifically and intensively interacted with currently known effective genes of pCRT, involving in DNA replication, cell cycle and DNA repair. In contrast, after excluding the cancer-related pCRT-response genes, the other DE genes between non-responders and responders were enriched in many pathways of drug and protein metabolisms and transports, and interacted with both the known effective genes and pharmacokinetic genes. Hence, these two types of DE genes should be distinguished for investigating mechanisms of pCRT response in LARCs.

Currently, neoadjuvant preoperation chemoradiation (pCRT) with 5-fluorouracil (5-FU)-based regimens followed by surgical resection is extensively employed for the locally advanced rectal cancers (LARCs)^1^. Usually, about 10–25% of LARCs have pathologic complete response, whereas most patients cannot benefit from the therapy but suffer delayed toxicity risk^2^. Although many factors mitigating the pCRT response are known, we are still unable to identify patients who will be susceptible to pCRT and benefit from this therapy^3^. Because response to pCRT correlates to positive survival outcomes^4^, it is necessary to investigate the mechanisms of pCRT response in LARCs. A common approach for investigating mechanisms of pCRT response is firstly identifying differentially expressed (DE) genes between the non-responders and responders^5^,^6^,^7^,^8^,^9^,^10^,^11^,^12^,^13^,^14^,^15^,^16^,^17^. However, such DE genes may have various origins. Some of such DE genes might be relevant to LARCs but deregulated with different patterns in non-responders and responders compared with normal rectal tissues. The other of such DE genes might be deregulated in neither non-responders nor responders, compared with normal rectal tissues, and thus be irrelevant to LARCs but simply reflect the pharmacokinetic differences between the non-responders and responders. Thus, it should be of great interest to clarify the different origins and roles of DE genes between the non-responders and responders.

However, it is difficult to identify DE genes in responders and/or non-responders compared with normal controls because currently few data for studying LARCs response to chemoradiation include normal rectal samples. Although there are some available data of normal rectal samples generated by different laboratories, they cannot be compared directly due to experimental batch effects^18^,^19^ and current methods for adjusting batch effects could distort biological signals^20^. Recently, we have reported an interesting biological phenomenon that the within-sample relative expression orderings (REOs) of genes within each of a particular type of normal tissues are highly stable but widely disrupted in the corresponding cancer tissues^21^. Based on that, *RankComp* was proposed to detect the deregulated genes in a disease sample through comparing the REOs in this sample with the stable REOs in normal human tissues^21^. Because all comparisons of REOs occur within individual samples, which obviates the need of data normalization, we will be able to utilize multiple datasets for the rectal normal samples to identify deregulated genes in cancer samples at the individual level.

In this study, using data of 34 normal rectal tissue samples from three datasets, we extracted gene pairs with identical REOs in all the samples as the landscape of normal rectal tissue. Then, we used *RankComp* to identify DE genes for each of 38 non-responders and 34 responders of LARCs. Subsequently, we identified 186 genes that had significantly different deregulation frequencies between the non-responders and responders, defined as cancer-related pCRT-response genes. Among these genes, 57 genes were shared with DE genes between the non-responders and responders identified with RankProduct^22^. After excluding the cancer-related pCRT-response genes, the other DE genes between the non-responders and responders were defined as cancer-unrelated pCRT-response genes. Finally, by pathway enrichment and protein-protein inteaction analyses, we found that the cancer-related and cancer-unrelated pCRT-response genes tend to play different roles in mechanisms of chemoradiation resistance. The framework of our research is described in Fig. 1.

## Results

### Cancer-related pCRT-response genes

Because only a limited number of normal rectal samples measured by a particular gene expression profiling platform could be obtained, we collected 34 normal rectal samples from three datasets measured by the Affymetrix, Illumina and Agilent platforms (see Methods), respectively, to construct cross-platform stable relative expression orderings (REOs) of gene pairs in normal rectal tissues. As reported in our recent paper^23^, based on gene pairs with stable REOs across normal samples measured by several platforms, RankComp could accurately detect DE genes in individual cancer samples measured by any of these platforms. Firstly, we evaluated the stability of REOs using a set of 21 normal tissue samples from GSE68204 measured by the Agilent platform and another set of 13 samples from two datasets (GSE9254 and GSE75548 measured by Affymetrix and Illumina platforms, respectively). In each set, we identified gene pairs which showed identical REOs in all the normal samples. A total 37,811,288 gene pairs were identified in both of the two sample sets, among which 92.9% had the same REO patterns across the two sets of samples. This was highly unlikely to happen by chance (Binomial test, *p*-value = 1.0E-100), suggesting that the REOs of these gene pairs are highly stable in normal rectal tissues measured by the three different platform. Thus, taking the 35,127,930 gene pairs with identical REOs in the 34 normal rectal tissue samples measured by the three platforms as the normal REOs landscape of rectal tissues, we could apply RankComp to detect DE genes in rectal cancer samples measured by any of these platforms^23^.

Then, we identified DE genes in each of the 72 samples of rectal cancer collected from two datasets (GSE35452 and GSE53781) with *RankComp* through comparing with the stable REO normal landscape of rectal tissue^21^ (see Methods). Averagely, 1596 DE genes per sample were identified, among which 186 genes had significantly different deregulation frequencies between the non-responders and responders (Fisher exact test, FDR < 0.05). These 186 genes, referred to as cancer-related pCRT-response genes, were significantly enriched in cancer-related pathways including DNA replication^24^, cell cycle^25^ and mismatch repair^26^ (Hypergeometric distribution model, FDR < 0.2). The pathways were discribed in Table 1. Notably, we found no significant pathways with a stricter FDR control level of 0.05, possibly due to the insufficient power of pathway enrichment analysis based on only 186 cancer-related pCRT-response genes found in the data. Thus, we detected significant pathways with a loose FDR control level of 0.2 to provide potentially functional clues of the cancer-related pCRT-response genes.

### Cancer-unrelated pCRT-response genes

Firstly, we identified 1288 and 805 DE genes between the non-responders and responders in the GSE35452 and GSE53781 datasets, respectively (RankProduct, FDR < 5%). The two lists of DE genes shared 101 genes, of which 80% showed consistent deregulation directions (up- or down-regulations) in the non-responders compared with responders across the two datasets. This consistency score was unlikely to be observed by random chance(Binomial distribution test, *p*-value = 1.3E-10), indicating that at least the overlaps of the two lists of DE genes between the non-responders and responders were significantly reproducible in the two datasets. Notably, the percentage of overlapping genes between the two lists of DE genes was apparently low, which indicated that only a small percentage of DE genes could be found in each of the two datasets due to insufficient statistical power^27^,^28^.

Considering the insufficient power of DE genes detection, we merged the two lists of DE genes, excluding inconsistent DE genes between the two datasets, and obtained a list of 1976 DE genes. These 1976 DE genes, referred to as pCRT-response genes for simplicity, included 57 of the cancer-related pCRT-response genes (as shown in Supplementary Table 2). After excluding these 57 genes, the other 1919 DE genes were defined as the cancer-unrelated pCRT-response genes, which were significantly enriched in various metabolic pathways including metabolism of xenobiotics by cytochrome P450^29^, and other pathways including oxidative phosphorylation and extracellular matrix receptor interaction (Hypergeometric distribution model, FDR < 0.2). The pathways were shown in Table 1.

### PPI network analysis of the two types of pCRT-response genes

We collected 113 genes known to be involved in LARCs response to pCRT, including 28 genes participating in metabolisms and transports of drug, 47 genes participating in purine and pyrimidine metabolism, 17 downstream effective genes of 5-FU which play roles in DNA repair, cell cycle arrest and apoptosis^30^ and 21 radio-response genes playing roles in DNA-damage related function^31^. Because the metabolism genes of purine and pyrimidine participate in DNA-damage-related function^30^, we classified them as effective genes together with the downstream effective genes of 5-FU and the radio-response genes, including a total 85 genes. The other 28 genes participating in drug metabolism and transports were referred to as the pharmacokinetic genes. The effective genes and pharmacokinetics genes are described in Supplementary Table 1.

**Figure 2:**
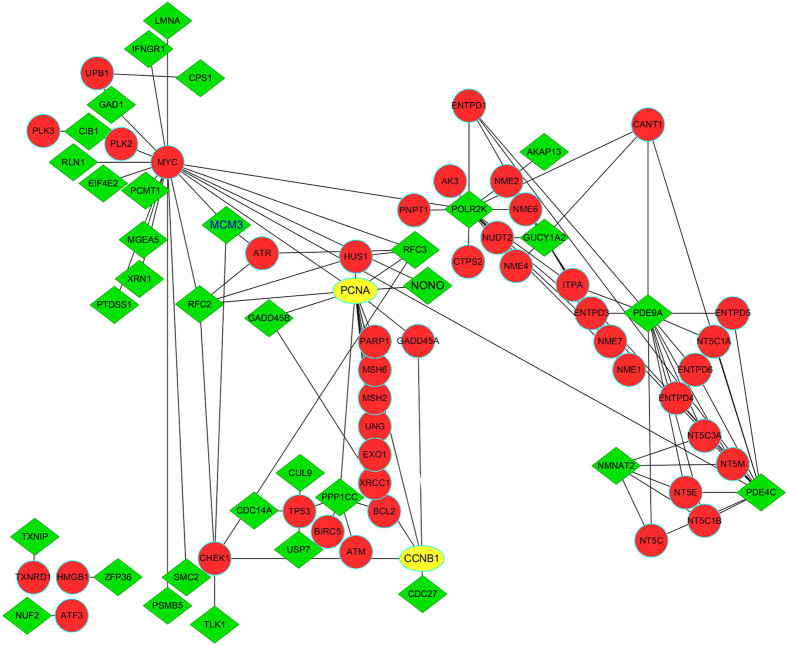
The direct PPI links between the cancer-related pCRT-response genes and the known effective genes of pCRT. The red (circular) nodes denote the known effective genes of pCRT. The green (diamond-shaped) nodes denote the cancer-related pCRT-response genes. The yellow (oval) nodes denote the genes overlapped between the cancer-related pCRT-response genes and the known effective genes of pCRT. MCM3, detected as a cancer-related pCRT-response gene, interacts with MYC, CHEK1 and ATR.

Then, through the human protein-protein interaction (PPI) network^32^, we showed that cancer-related pCRT-response genes tend to closely connected with the effective genes only, whereas the cancer-unrelated pCRT-response genes tend to interact with the pharmacokinetic genes as well as the effective genes. To be more specific, for the 186 cancer-related and 1919 cancer-unrelated pCRT-response genes defined in the above section, 124 and 1405 genes were mapped in the human PPI network, respectively. The 124 cancer-related pCRT-response genes had 117 and zero direct interactions with the effective genes and pharmacokinetic genes, respectively (See Fig. 2). And the 1405 cancer-unrelated pCRT-response genes had 672 and 54 direct interactions with the effective genes and the pharmacokinetic genes, respectively. Notably, the average number of the direct interactions between a cancer-related pCRT-response gene and the effective genes was 0.94, which was significantly more than the corresponding average number of 0.48 for the 1405 cancer-unrelated pCRT-response genes (Rank sum test, *p*-value = 2.2E-2).

## Discussion

In this work, we proposed to distinguish cancer-related and cancer-unrelated pCRT-response genes for genes differentially expressed between non-responders and responders of LARCs. We showed that these two types of genes affect LARCs response to pCRT in totally different ways. Notably, according to the framework of cancer hallmarks network^33^, the pathways enriched with the cancer-related pCRT-response genes are related to the survival network which presents the cancer traits of resistance to cell death, sustaining chronic proliferation and blocking signals of inhibitory growth. Furthermore, PPI network analyses revealed that the cancer-related pCRT-response genes specifically and intensively interact with the known effective genes of pCRT, mostly conducting the functions in well known pCRT-response related pathways including DNA replication, cell cycle and DNA repair^30^,^31^. For example, MCM3 was found as a cancer-related pCRT-response gene and it interacts with MYC, CHEK1 and ATR, which are all the known effective genes of pCRT-response^30^,^31^ (See Fig. 2).

**Figure 3:**
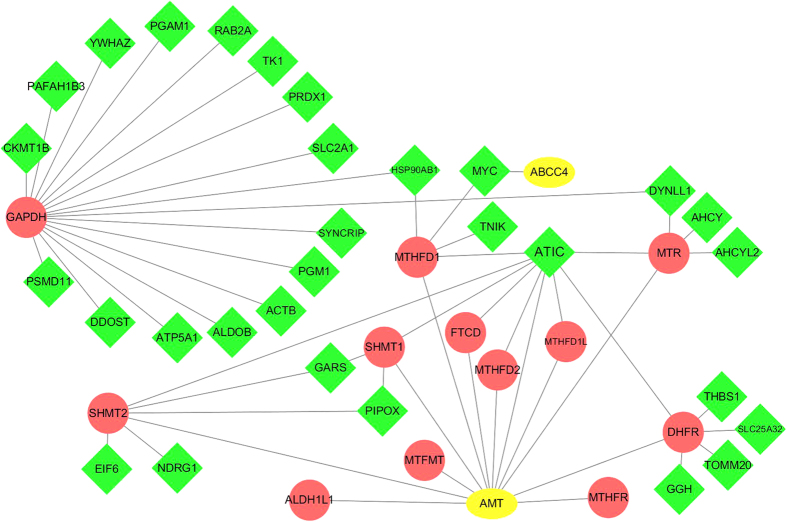
The direct PPI links between the cancer-unrelated pCRT-response genes and the known pharmacokinetics genes of 5-FU. The red (circular) nodes denote the known pharmacokinetics genes of 5-FU. The green (diamond-shaped) nodes denote the cancer-unrelated pCRT-response genes. The yellow (oval) nodes denote the genes overlapped between the cancer-unrelated pCRT-response genes and the known pharmacokinetics genes of 5-FU. ATIC, detected as a cancer-unrelated pCRT-response gene, has the largest number of interaction links with the known pharmacokinetics genes of 5-FU.

In contrast, the cancer-unrelated pCRT-response genes were significantly enriched in typical metabolism pathways related to drug metabolisms such as cytochrome P450 which contributes to multidrug resistance in tumor^34^ and other diseases^35^. Outstandingly, we found that the cancer-unrelated pCRT-response genes tend to interact with the known pharmacokinetic genes (See Fig. 3). Together, these results suggested that the cancer-unrelated pCRT-response genes may determine the metabolism characteristics to shape LARCs response. For example, ATIC was detected as a cancer-unrelated pCRT-response gene and it interacts with MTR, AMT, MTHFD1/2, SHMT1/2, FTCD and DHFR, which are all involved in the inhibition of thymidylate synthase^30^,^31^ (See Fig. 3). And genetic variant of ATIC has been confirmed to be a pharmacokinetics marker of methotrexate^36^. On the other hand, our results showed that the cancer-unrelated pCRT-response genes also interact with effective genes of pCRT. However, this result should be explained carefully because some of the cancer-unrelated pCRT-response genes could be mistakenly identified due to the insufficient power of the identification of cancer-related pCRT-response genes. The number of available samples of normal rectal tissues was only 34, which could be insufficient to detect all the genes pairs with stable REOs in normal rectal tissues and the individual-level DE genes for each LARC. Therefore, it is important that the cancer-related pCRT-response genes should be further studied using larger samples of normal and LARCs.

In summary, the two types of pCRT-response genes should be distinguished in studying mechanisms of LARCs response to pCRT.

## Materials and Methods

### Data and pre-processing

Five microarray datasets for rectal normal samples and LARCs were downloaded from Gene Expression Omnibus^37^ (GEO, http://www.ncbi.nlm.nih.gov/geo/), as described in detail in Table 2 and 3. For the data from GSE9254 and GSE35452 measured by the Affymetrix platform, the raw data was processed for background adjustment via the Robust Multi-array Average algorithm^38^ without quantile normalization because all comparisons of gene relative orderings occurred within individual samples. For the data derived from GSE68204 measured by the Agilent platform, the raw fluorescent signal intensity values of the channel (gMedianSignal or rMedianSignal) for normal samples minus the corresponding background signal intensities as the probe-expression matrix. For the data of GSE53781 and GSE75548 measured by CodeLink bioarrays and Illumina platform, we directly downloaded the processed data. Then, each probe-set ID was mapped to its Entrez gene ID with the corresponding custom CDF files. If several probesets were mapped to a gene, the expression value for the gene was defined as the arithmetic mean of the values of the multiple probesets (on the log2 scale).

For the data of LARCs extracted from GSE35452 and GSE53781, there were some subtle differences in the protocol of chemoradiation and tumor response assessment. Firstly, the 5-fluorouracil-based regimens of chemotherapy in the two studies were ftorafur-uracil and capecitabine, respectively, both of which are metabolized to 5-FU *in vivo*^39^. Secondly, tumor regression grade (TRG) systems adopted in the two studies were Mandard and AJCC (American Joint Committee on Cancer) systems^7^,^16^, respectively. According to the Mandard system, patients with TRG 1 and 2 scores are classified as responders, and patients with TRG 3 to 5 scores were classified as nonresponders. According to the AJCC system, patients with TRG 0 and 1 scores were classified as responders, and patients with TRG 2 and 3 scores were classified as nonresponders. However, the difference between the response classification of the two scoring systems is small^40^.

### Identification of the cancer-related pCRT-response genes

The threshold value of frequency for stable relative ordering of gene pair in normal rectal samples was defined as 100%.

Then, we applied the RankComp algorithm^21^ to identify differentially expressed genes (DE genes) at the individual level. Briefly, to determine whether a given gene *X* is differentially expressed in a given disease sample, Fisher’s exact test is used to test the null hypothesis that the frequency of reversal gene pairs supporting its upregulation and the frequency of reversal gene pairs supporting its downregulation are not significantly different. If the expression level of gene *X* is consistently lower (or higher) than another gene in normal samples but reversal in a disease sample, then this reversal gene pair is considered to support upregulation (or downregulation) of gene *X* in this disease sample. Finally, a filtering process is utilized to retain only those DE genes which are still significant after excluding their coupled reversal gene pairs including any other DE genes. The detail of this method is discribed in our previous work^21^. The *p*-values were adjusted using the Benjamini and Hochberg procedure to control the False Discovery Rate^41^. Genes that were dysregulated in a non-randomly high percentage of cancer samples were defined as cancer-related genes based on the binomial test with p < 0.05.

For a specific gene, the Fisher’s exact test was used to test whether its deregulation frequency in the non-responders is significantly different from that in the responders. The *p*-values were adjusted using the Benjamini and Hochberg procedure to control the False Discovery Rate.

### Identification of the DE gene between non-responders and responders

We used the RankProd algorithm^22^ to identify DE genes between the responders and non-responders. The *p*-values were adjusted using the Benjamini and Hochberg procedure^41^ to control the False Discovery Rate.

### Consistence evaluation of two DE gene lists

For DE genes from two independent datasets sharing *k* DE genes, of which *s* genes had the same up- or down-regulation directions, the concordance score was calculated as *s/k*. This score was used to evaluate the consistence of DE genes extracted from independent datasets. The probability of observing a concordance score(*s/k*.) by chance can be evaluated using the cumulative binomial distribution model as follows:


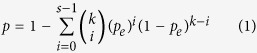


In Equation (1), *p*_*e*_ is the probability of a gene having the same dysregulation direction in two gene lists by random chance (here, *p*_*e*_ = 0.5). The concordance score is considered significantly if the *p*-value < 0.05.

### Functional enrichment analysis

Data of 223 pathways covering 6290 unique genes were extracted from the Kyoto Encyclopedia of Genes and Genomes (KEGG)^42^ on 10 May 2015. The hypergeometric distribution model, or equally the one-tailed Fisher exact test, was used to determine the significance of biological pathways enriched with the cancer-related and cancer-unrelated pCRT-response genes, respectively. The *p*-values were adjusted using the Benjamini and Hochberg procedure^41^.

### Human protein-protein interaction (PPI) analysis

The PPI data integrating eight databases were composed of 142, 583 distinct interactions involving 13693 human proteins, which were discribed in our previous work^32^.

Wilcoxon’s rank-sum test was used to test whether the number of interaction links between the known pCRT-response genes and a gene set were significantly different from the number of interaction links between the known pCRT-response genes and another gene set.

### Statistical software for analysis

All statistical analyses were performed using the R 3.12 (http://www.r-project.org/).

## Additional Information

**How to cite this article**: Guo, Y. *et al.* Discriminating cancer-related and cancer-unrelated chemoradiation-response genes for locally advanced rectal cancers. *Sci. Rep.* **6**, 36935; doi: 10.1038/srep36935 (2016).

**Publisher’s note**: Springer Nature remains neutral with regard to jurisdictional claims in published maps and institutional affiliations.

## Supplementary Material

Supplementary Information

## Figures and Tables

**Figure 1:**
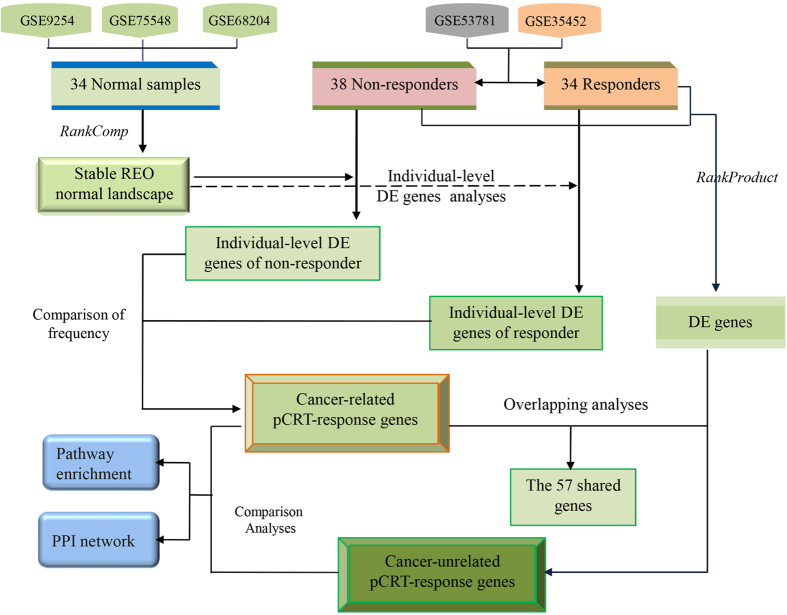
The flowchart of the analysis procedure. DE genes between the non-responders and responders includes 57 cancer-related pCRT-response genes. After excluding these 57 genes, the other DE genes between the non-responders and responders were defined as the cancer-unrelated pCRT-response genes.

**Table 1:** The pathways enriched with cancer-related and cancer-unrelated pCRT-response genes, respectively.

Genes of pCRT-response	KEGG Pathway	P-value
Cancer-related	DNA replication	5.24E-04
	Mismatch repair	1.52E-03
	Cell cycle	1.80E-03
Cancer-unrelated	Metabolism of xenobiotics by cytochrome P450	9.45E-03
	Cysteine and methionine metabolism	2.74E-03
	Metabolic pathways	2.32E-03
	Ribosome	1.72E-08
	Proteasome	9.74E-07
	Protein digestion and absorption	4.42E-07
	ECM-receptor interaction	1.65E-04
	Oxidative phosphorylation	1.53E-08

**Table 2:** The data of normal rectal samples used for identifying stable gene pairs.

Accession number	Platforms	Number of genes	Number of samples	References (PMID)
GSE68204	Agilent-014850 Whole Human Genome Microarray	19596	21	27225591
GSE9254	Affymetrix Human Genome U133 Plus 2.0	20283	7	18056783
GSE75548	Illumina HumanHT-12 V4.0 expression beadchip	30500	6	26911399

**Table 3:** The data of LARCs samples used to identify the cancer-related pCRT-response genes.

Accession number	Platforms	Number of genes	Number of non-responders	Number of responders	References (PMID)
GSE35452	Affymetrix Human Genome U133 Plus 2.0	20283	22	24	16585155
GSE53781	CodeLink Human Whole Genome Array	13165	16	10	25380052

## References

[b1] Gollins S. & Sebag-Montefiore D. Neoadjuvant Treatment Strategies for Locally Advanced Rectal Cancer. Clinical oncology 28, 146–151 (2016). 26645661 10.1016/j.clon.2015.11.003

[b2] Smith J. J. & Garcia-Aguilar J. Advances and challenges in treatment of locally advanced rectal cancer. Journal of clinical oncology 33, 1797–1808 (2015). 25918296 10.1200/JCO.2014.60.1054 PMC4559608

[b3] Kim N. K. & Hur H. New Perspectives on Predictive Biomarkers of Tumor Response and Their Clinical Application in Preoperative Chemoradiation Therapy for Rectal Cancer. Yonsei medical journal 56, 1461–1477 (2015). 26446626 10.3349/ymj.2015.56.6.1461 PMC4630032

[b4] Park I. J. *et al.* Neoadjuvant treatment response as an early response indicator for patients with rectal cancer. Journal of clinical oncology 30, 1770–1776 (2012). 22493423 10.1200/JCO.2011.39.7901 PMC3383178

[b5] Conde-Muino R. *et al.* Predictive Biomarkers to Chemoradiation in Locally Advanced Rectal Cancer. BioMed research international 2015, 921435 (2015). 26504848 10.1155/2015/921435 PMC4609421

[b6] Ghadimi B. M. *et al.* Effectiveness of gene expression profiling for response prediction of rectal adenocarcinomas to preoperative chemoradiotherapy. Journal of clinical oncology 23, 1826–1838 (2005). 15774776 10.1200/JCO.2005.00.406 PMC4721601

[b7] Watanabe T. *et al.* Prediction of sensitivity of rectal cancer cells in response to preoperative radiotherapy by DNA microarray analysis of gene expression profiles. Cancer research 66, 3370–3374 (2006). 16585155 10.1158/0008-5472.CAN-05-3834

[b8] Kim I. J. *et al.* Microarray gene expression profiling for predicting complete response to preoperative chemoradiotherapy in patients with advanced rectal cancer. Diseases of the colon and rectum 50, 1342–1353 (2007). 17665260 10.1007/s10350-007-277-7

[b9] Ojima E., Inoue Y., Miki C., Mori M. & Kusunoki M. Effectiveness of gene expression profiling for response prediction of rectal cancer to preoperative radiotherapy. Journal of gastroenterology 42, 730–736 (2007). 17876542 10.1007/s00535-007-2089-x

[b10] Rimkus C. *et al.* Microarray-based prediction of tumor response to neoadjuvant radiochemotherapy of patients with locally advanced rectal cancer. Clinical gastroenterology and hepatology 6, 53–61 (2008). 18166477 10.1016/j.cgh.2007.10.022

[b11] Snipstad K. *et al.* New specific molecular targets for radio-chemotherapy of rectal cancer. Molecular oncology 4, 52–64 (2010). 19969511 10.1016/j.molonc.2009.11.002 PMC5527962

[b12] Brettingham-Moore K. H. *et al.* Pretreatment transcriptional profiling for predicting response to neoadjuvant chemoradiotherapy in rectal adenocarcinoma. Clinical cancer research 17, 3039–3047 (2011). 21224373 10.1158/1078-0432.CCR-10-2915

[b13] Nishioka M. *et al.* Gene expression profile can predict pathological response to preoperative chemoradiotherapy in rectal cancer. Cancer genomics & proteomics 8, 87–92 (2011). 21471518

[b14] Gantt G. A. *et al.* Gene expression profile is associated with chemoradiation resistance in rectal cancer. Colorectal disease 16, 57–66 (2014). 24034224 10.1111/codi.12395

[b15] Watanabe T. *et al.* Prediction of response to preoperative chemoradiotherapy in rectal cancer by using reverse transcriptase polymerase chain reaction analysis of four genes. Diseases of the colon and rectum 57, 23–31 (2014). 24316942 10.1097/01.dcr.0000437688.33795.9d

[b16] Palma P. *et al.* Expression profiling of rectal tumors defines response to neoadjuvant treatment related genes. PloS one 9, e112189 (2014). 25380052 10.1371/journal.pone.0112189 PMC4224421

[b17] Ryan J. E. *et al.* Predicting pathological complete response to neoadjuvant chemoradiotherapy in locally advanced rectal cancer: a systematic review. Colorectal disease 18, 234–246 (2016). 26531759 10.1111/codi.13207

[b18] Leek J. T. *et al.* Tackling the widespread and critical impact of batch effects in high-throughput data. Nature reviews. Genetics 11, 733–739 (2010). 10.1038/nrg2825 PMC3880143 20838408

[b19] Lazar C. *et al.* Batch effect removal methods for microarray gene expression data integration: a survey. Briefings in bioinformatics 14, 469–490 (2013). 22851511 10.1093/bib/bbs037

[b20] Wang D. *et al.* Extensive increase of microarray signals in cancers calls for novel normalization assumptions. Computational biology and chemistry 35, 126–130 (2011). 21704257 10.1016/j.compbiolchem.2011.04.006

[b21] Wang H. *et al.* Individual-level analysis of differential expression of genes and pathways for personalized medicine. Bioinformatics 31, 62–68 (2015). 25165092 10.1093/bioinformatics/btu522

[b22] Breitling R., Armengaud P., Amtmann A. & Herzyk P. Rank products: a simple, yet powerful, new method to detect differentially regulated genes in replicated microarray experiments. FEBS letters 573, 83–92 (2004). 15327980 10.1016/j.febslet.2004.07.055

[b23] Guan Q. *et al.* Differential expression analysis for individual cancer samples based on robust within-sample relative gene expression orderings across multiple profiling platforms. Oncotarget, doi: 10.18632/oncotarget.11996 (2016). PMC5356599 27634898

[b24] Liu B. *et al.* Mechanisms of mutagenesis: DNA replication in the presence of DNA damage. Mutation research. Reviews in mutation research 768, 53–67 (2016). 27234563 10.1016/j.mrrev.2016.03.006 PMC5237373

[b25] Williams G. H. & Stoeber K. The cell cycle and cancer. The Journal of pathology 226, 352–364 (2012). 21990031 10.1002/path.3022

[b26] Bellizzi A. M. & Frankel W. L. Colorectal cancer due to deficiency in DNA mismatch repair function: a review. Advances in anatomic pathology 16, 405–417 (2009). 19851131 10.1097/PAP.0b013e3181bb6bdc

[b27] Zhang M. *et al.* Apparently low reproducibility of true differential expression discoveries in microarray studies. Bioinformatics 24, 2057–2063 (2008). 18632747 10.1093/bioinformatics/btn365

[b28] Zhang M. *et al.* Evaluating reproducibility of differential expression discoveries in microarray studies by considering correlated molecular changes. Bioinformatics 25, 1662–1668 (2009). 19417058 10.1093/bioinformatics/btp295 PMC2940240

[b29] Olsen L., Oostenbrink C. & Jorgensen F. S. Prediction of cytochrome P450 mediated metabolism. Advanced drug delivery reviews 86, 61–71 (2015). 25958010 10.1016/j.addr.2015.04.020

[b30] Tan W. L. *et al.* Low cytosine triphosphate synthase 2 expression renders resistance to 5-fluorouracil in colorectal cancer. Cancer biology & therapy 11, 599–608 (2011). 21378502 10.4161/cbt.11.6.14670

[b31] Oh J. H. & Deasy J. O. Inference of radio-responsive gene regulatory networks using the graphical lasso algorithm. BMC bioinformatics 15 Suppl 7, S5 (2014). 10.1186/1471-2105-15-S7-S5 PMC4110733 25077716

[b32] Tong M. *et al.* Multi-omics landscapes of colorectal cancer subtypes discriminated by an individualized prognostic signature for 5-fluorouracil-based chemotherapy. Oncogenesis 5, e242, doi: 10.1038/oncsis.2016.51 (2016). 27429074 PMC5399173

[b33] Wang E. *et al.* Predictive genomics: a cancer hallmark network framework for predicting tumor clinical phenotypes using genome sequencing data. Seminars in cancer biology 30, 4–12 (2015). 24747696 10.1016/j.semcancer.2014.04.002

[b34] Mittal B., Tulsyan S., Kumar S., Mittal R. D. & Agarwal G. Cytochrome P450 in Cancer Susceptibility and Treatment. Advances in clinical chemistry 71, 77–139 (2015). 26411412 10.1016/bs.acc.2015.06.003

[b35] Hodgson K. *et al.* Genetic differences in cytochrome P450 enzymes and antidepressant treatment response. Journal of psychopharmacology 28, 133–141 (2014). 24257813 10.1177/0269881113512041

[b36] Salazar J. *et al.* Polymorphisms in genes involved in the mechanism of action of methotrexate: are they associated with outcome in rheumatoid arthritis patients? Pharmacogenomics 15, 1079–1090 (2014). 25084201 10.2217/pgs.14.67

[b37] Edgar R., Domrachev M. & Lash A. E. Gene Expression Omnibus: NCBI gene expression and hybridization array data repository. Nucleic acids research 30, 207–210 (2002). 11752295 10.1093/nar/30.1.207 PMC99122

[b38] Irizarry R. A. *et al.* Exploration, normalization, and summaries of high density oligonucleotide array probe level data. Biostatistics 4, 249–264 (2003). 12925520 10.1093/biostatistics/4.2.249

[b39] Bennouna J., Saunders M. & Douillard J. Y. The role of UFT in metastatic colorectal cancer. Oncology 76, 301–310 (2009). 19299903 10.1159/000209334

[b40] Kim S. H. *et al.* What Is the Ideal Tumor Regression Grading System in Rectal Cancer Patients after Preoperative Chemoradiotherapy? Cancer research and treatment 48, 998–1009 (2016). 26511803 10.4143/crt.2015.254 PMC4946373

[b41] Benjamini Y., Drai D., Elmer G., Kafkafi N. & Golani I. Controlling the false discovery rate in behavior genetics research. Behavioural brain research 125, 279–284 (2001). 11682119 10.1016/s0166-4328(01)00297-2

[b42] Kanehisa M., Goto S., Sato Y., Furumichi M. & Tanabe M. KEGG for integration and interpretation of large-scale molecular data sets. Nucleic acids research 40, D109–D114 (2012). 22080510 10.1093/nar/gkr988 PMC3245020

