# Supplementary material for: Discriminating cancer-related and cancer-unrelated chemoradiation-response genes for locally advanced rectal cancers
Source: Sci Rep. 2016 Nov 15;6:36935. doi: 10.1038/srep36935 (PMC5109405; doi:10.1038/srep36935)
Supplement: Supplementary Information [file srep36935-s1.pdf]

Discriminating cancer-related and cancer-unrelated chemoradiation-response genes for locally advanced rectal cancers

You Guo[1,2], Jun Cheng[1], Lu Ao[1], Xiangyu Li[1], Qingzhou Guan[1], Juan Zhang[1], Haidan Yan[1], Hao Cai[1], Qiao Gao[3], Weizhong Jiang[3]*, Zheng Guo[1]*

Supplementary Information

Supplementary Table 1. The known 113 genes of pCRT-response including 85 effective genes and 28 pharmacokinetic genes, which were collected from the two previous study[1,2].

Supplementary Table 2. The 57 genes shared by cancer-related pCRT-response genes and differentially expressed (DE) genes between the non-responders and responders of LARCs.

Supplementary Tables

Supplementary Table 1. The known113 genes involved in pCRT-response including 85 effective genes and 28 pharmacokinetic genes.

Gene ID	Gene symbol	Functional category	Function
318	NUDT2	effective	Metabolism of pyrimidine and purine
471	ATIC	effective	Metabolism of pyrimidine and purine
953	ENTPD1	effective	Metabolism of pyrimidine and purine
955	ENTPD6	effective	Metabolism of pyrimidine and purine
956	ENTPD3	effective	Metabolism of pyrimidine and purine
957	ENTPD5	effective	Metabolism of pyrimidine and purine
978	CDA	effective	Metabolism of pyrimidine and purine
1806	DPYD	effective	Metabolism of pyrimidine and purine
1807	DPYS	effective	Metabolism of pyrimidine and purine
1841	DTYMK	effective	Metabolism of pyrimidine and purine
1854	DUT	Effective	Metabolism of pyrimidine and purine
1890	TYMP	effective	Metabolism of pyrimidine and purine
2618	GART	effective	Metabolism of pyrimidine and purine
3704	ITPA	effective	Metabolism of pyrimidine and purine
4830	NME1	effective	Metabolism of pyrimidine and purine
4831	NME2	effective	Metabolism of pyrimidine and purine
4832	NME3	effective	Metabolism of pyrimidine and purine
4833	NME4	effective	Metabolism of pyrimidine and purine
4907	NT5E	effective	Metabolism of pyrimidine and purine
6240	RRM1	effective	Metabolism of pyrimidine and purine
6241	RRM2	effective	Metabolism of pyrimidine and purine
7083	TK1	effective	Metabolism of pyrimidine and purine
7084	TK2	effective	Metabolism of pyrimidine and purine
7296	TXNRD1	effective	Metabolism of pyrimidine and purine
7298	TYMS	effective	Metabolism of pyrimidine and purine

7371	UCK2	effective	Metabolism of pyrimidine and purine
7372	UMPS	effective	Metabolism of pyrimidine and purine
7378	UPP1	effective	Metabolism of pyrimidine and purine
8382	NME5	effective	Metabolism of pyrimidine and purine
9583	ENTPD4	effective	Metabolism of pyrimidine and purine
10201	NME6	effective	Metabolism of pyrimidine and purine
10587	TXNRD2	effective	Metabolism of pyrimidine and purine
22978	NT5C2	effective	Metabolism of pyrimidine and purine
29922	NME7	effective	Metabolism of pyrimidine and purine
30833	NT5C	effective	Metabolism of pyrimidine and purine
50484	RRM2B	effective	Metabolism of pyrimidine and purine
50808	AK3	effective	Metabolism of pyrimidine and purine
51251	NT5C3	effective	Metabolism of pyrimidine and purine
51733	UPB1	effective	Metabolism of pyrimidine and purine
56474	CTPS2	effective	Metabolism of pyrimidine and purine
56953	NT5M	effective	Metabolism of pyrimidine and purine
83549	UCK1	effective	Metabolism of pyrimidine and purine
84618	NT5C1A	effective	Metabolism of pyrimidine and purine
87178	PNPT1	effective	Metabolism of pyrimidine and purine
93034	NT5C1B	effective	Metabolism of pyrimidine and purine
124583	CANT1	effective	Metabolism of pyrimidine and purine
151531	UPP2	effective	Metabolism of pyrimidine and purine
59	ACTA2	effective	DNA repair/apoptosis/cell cycle regulation
142	PARP1	effective	DNA repair /apoptosis/cell cycle regulation
332	BIRC5	effective	DNA repair/apoptosis/cell cycle regulation
467	ATF3	effective	DNA repair/apoptosis/cell cycle regulation
472	ATM	effective	DNA repair/apoptosis/cell cycle regulation
545	ATR	effective	DNA repair/apoptosis/cell cycle regulation
581	BAX	effective	DNA repair/apoptosis/cell cycle regulation

596	BCL2	effective	DNA repair/apoptosis/cell cycle regulation
891	CCNB1	effective	DNA repair/apoptosis/cell cycle regulation
900	CCNG1	effective	DNA repair/apoptosis/cell cycle regulation
970	CD70	effective	DNA repair/apoptosis/cell cycle regulation
1111	CHEK1	effective	DNA repair/apoptosis/cell cycle regulation
1263	PLK3	effective	DNA repair/apoptosis/cell cycle regulation
1643	DDB2	effective	DNA repair/apoptosis/cell cycle regulation
1647	GADD45A	effective	DNA repair/apoptosis/cell cycle regulation
2232	FDXR	effective	DNA repair/apoptosis/cell cycle regulation
2956	MSH6	effective	DNA repair/apoptosis/cell cycle regulation
3146	HMGB1	effective	DNA repair/apoptosis/cell cycle regulation
3364	HUS1	effective	DNA repair/apoptosis/cell cycle regulation
4292	MLH1	effective	DNA repair/apoptosis/cell cycle regulation
4318	MMP9	effective	DNA repair/apoptosis/cell cycle regulation
4436	MSH2	effective	DNA repair/apoptosis/cell cycle regulation
4609	MYC	effective	DNA repair/apoptosis/cell cycle regulation
5111	PCNA	effective	DNA repair/apoptosis/cell cycle regulation
5395	PMS2	effective	DNA repair/apoptosis/cell cycle regulation
7157	TP53	effective	DNA repair/apoptosis/cell cycle regulation
7374	UNG	effective	DNA repair/apoptosis/cell cycle regulation
7515	XRCC1	effective	DNA repair/apoptosis/cell cycle regulation
8493	PPM1D	effective	DNA repair/apoptosis/cell cycle regulation
8795	TNFRSF10<br>8	effective	DNA repair/apoptosis/cell cycle regulation
9156	EXO1	effective	DNA repair/apoptosis/cell cycle regulation
9538	EI24	effective	DNA repair/apoptosis/cell cycle regulation
9656	MDC1	effective	DNA repair/apoptosis/cell cycle regulation
10038	PARP2	effective	DNA repair/apoptosis/cell cycle regulation
10769	PLK2	effective	DNA repair/apoptosis/cell cycle regulation

11200	CHEK2	effective	DNA repair/apoptosis/cell cycle regulation
23583	SMUG1	effective	DNA repair/apoptosis/cell cycle regulation
27113	BBC3	effective	DNA repair/apoptosis/cell cycle regulation
275	AMT	pharmacokinetic	Metabolism of drug
1066	CES1	pharmacokinetic	Metabolism of drug
1719	DHFR	pharmacokinetic	Metabolism of drug
2030	SLC29A1	pharmacokinetic	Transporters
2597	GAPDH	pharmacokinetic	Housekeeping
3177	SLC29A2	pharmacokinetic	Transporters
4522	MTHFD1	pharmacokinetic	Metabolism of drug
4524	MTHFR	pharmacokinetic	Metabolism of drug
4548	MTR	pharmacokinetic	Metabolism of drug
6470	SHMT1	pharmacokinetic	Metabolism of drug
6472	SHMT2	pharmacokinetic	Metabolism of drug
8824	CES2	pharmacokinetic	Metabolism of drug
9153	SLC28A2	pharmacokinetic	Transporters
9154	SLC28A1	pharmacokinetic	Transporters
10057	ABCC5	pharmacokinetic	Transporters
10257	ABCC4	pharmacokinetic	Transporters
10588	MTHFS	pharmacokinetic	Metabolism of drug
10797	MTHFD2	pharmacokinetic	Metabolism of drug
10840	ALDH1L1	pharmacokinetic	Metabolism of drug
10841	FTCD	pharmacokinetic	Metabolism of drug
25902	MTHFD1L	pharmacokinetic	Metabolism of drug
55315	SLC29A3	pharmacokinetic	Transporters
64078	SLC28A3	pharmacokinetic	Transporters
85320	ABCC11	pharmacokinetic	Transporters
94160	ABCC12	pharmacokinetic	Transporters
123263	MTFMT	pharmacokinetic	Metabolism of drug

222962	SLC29A4	pharmacokinetic	Transporters
401840	NP	pharmacokinetic	Metabolism of drug

Supplementary Table 2. The 57 genes shared by cancer-related pCRT-response genes and DE genes between the non-responders and responders of LARCs

Gene ID	Gene symbol	Results from DE genes	Results from the cancer-related pCRT-response genes	
		Subgroup with higher expression	Subgroup with higher deregulated frequency	Dysregulated direction
891	CCNB1 [1]	Responder	Responder	up-regulated
1160	CKMT2	Responder	Responder	up-regulated
1373	CPS1	Non-responder	Non-responder	up-regulated
1734	DIO2	Responder	Responder	up-regulated
1894	ECT2	Responder	Responder	up-regulated
2920	CXCL2	Responder	Responder	up-regulated
3207	HOXA11	Responder	Responder	up-regulated
3627	CXCL10	Responder	Responder	up-regulated
4285	MIPEP	Responder	Responder	up-regulated
4841	NONO [2]	Responder	Non-responder	down-regulated
5122	PCSK1	Non-responder	Non-responder	up-regulated
5152	PDE9A	Non-responder	Responder	down-regulated
5339	PLEC	Non-responder	Non-responder	up-regulated
5440	POLR2K	Responder	Responder	up-regulated
5501	PPP1CC	Responder	Non-responder	down-regulated
5693	PSMB5	Responder	Non-responder	down-regulated
5983	RFC3	Responder	Responder	up-regulated

6152	RPL24	Responder	Non-responder	down-regulated
6662	SOX9	Responder	Responder	up-regulated
6920	TCEA3	Non-responder	Responder	down-regulated
6947	TCN1	Non-responder	Non-responder	up-regulated
7538	ZFP36	Responder	Non-responder	down-regulated
8838	WISP3	Non-responder	Non-responder	up-regulated
9254	CACNA2D2	Non-responder	Non-responder	up-regulated
10383	TUBB4B	Responder	Non-responder	down-regulated
10418	SPON1	Non-responder	Responder	down-regulated
10449	ACAA2	Responder	Non-responder	down-regulated
10519	CIB1	Non-responder	Responder	down-regulated
10628	TXNIP	Non-responder	Responder	down-regulated
11075	STMN2	Non-responder	Responder	down-regulated
11346	SYNPO	Non-responder	Non-responder	up-regulated
23584	VSIG2	Non-responder	Responder	down-regulated
27166	PRELID1	Responder	Non-responder	down-regulated
27243	CHMP2A	Non-responder	Responder	down-regulated
29887	SNX10	Responder	Responder	up-regulated
30812	SOX8	Non-responder	Responder	down-regulated
51090	PLLP	Non-responder	Responder	down-regulated
51655	RASD1	Non-responder	Responder	down-regulated

54716	SLC6A20	Responder	Responder	up-regulated
54892	NCAPG2	Responder	Responder	up-regulated
55107	ANO1	Non-responder	Non-responder	up-regulated
55603	FAM46A	Non-responder	Responder	down-regulated
57002	C7orf36	Responder	Responder	up-regulated
57556	SEMA6A	Non-responder	Responder	down-regulated
79576	NKAP	Responder	Responder	up-regulated
79633	FAT4	Responder	Responder	up-regulated
83540	NUF2	Responder	Responder	up-regulated
83998	REG4	Non-responder	Non-responder	up-regulated
84133	ZNRF3	Responder	Responder	up-regulated
84141	FAM176A	Responder	Responder	up-regulated
90293	KLHL13	Non-responder	Non-responder	up-regulated
93010	B3GNT7	Non-responder	Responder	down-regulated
116154	PHACTR3	Responder	Responder	up-regulated
220134	SKA1	Responder	Responder	up-regulated
221656	KDM1B	Responder	Responder	up-regulated
256714	MAP7D2	Responder	Responder	up-regulated
729993	SHISA9	Non-responder	Non-responder	up-regulated

Note: [1] DE genes revealed that CCNB1was up-regulated in the responders vs nonresponders. In contrast, the cancer-related pCRT-response genes revealed that CCNB1 was up-regulated in LARCs vs normal samples, and the deregulated frequency of the responders was more than that of

the nonresponders. [2] DE genes revealed that NONO was up-regulated in the responders vs nonresponders. In contrast, the cancer-related pCRT-response genes revealed that NONO was down-regulated in LARCs vs normal samples, and the deregulated frequency of the responders was lower than that of the nonresponders. The difference of deregulated pattern between CCNB1 and NONO cannot be detected by DE genes.

References

1. Tan, W. L. *et al.* Low cytosine triphosphate synthase 2 expression renders resistance to 5-fluorouracil in colorectal cancer. *Cancer biology & therapy* **11**, 599-608 (2011).
2. Oh, J. H. & Deasy, J. O. Inference of radio-responsive gene regulatory networks using the graphical lasso algorithm. *BMC bioinformatics* **15 Suppl 7**, S5, doi:10.1186/1471-2105-15-S7-S5 (2014).
